# Cognitive Impairments in Occupational Burnout – Error Processing and Its Indices of Reactive and Proactive Control

**DOI:** 10.3389/fpsyg.2017.00676

**Published:** 2017-05-01

**Authors:** Krystyna Golonka, Justyna Mojsa-Kaja, Magda Gawlowska, Katarzyna Popiel

**Affiliations:** Institute of Applied Psychology, Jagiellonian UniversityKrakow, Poland

**Keywords:** burnout, cognitive impairments, error processing, ERN, Pe, reactive and proactive control

## Abstract

The presented study refers to cognitive aspects of burnout as the effects of long-term work-related stress. The purpose of the study was to investigate electrophysiological correlates of burnout to explain the mechanisms of the core burnout symptoms: exhaustion and depersonalization/cynicism. The analyzed error-related electrophysiological markers shed light on impaired cognitive mechanisms and the specific changes in information-processing in burnout. In the EEG study design (*N* = 80), two components of error-related potential (ERP), error-related negativity (ERN), and error positivity (Pe), were analyzed. In the non-clinical burnout group (*N* = 40), a significant increase in ERN amplitude and a decrease in Pe amplitude were observed compared to controls (*N* = 40). Enhanced error detection, indexed by increased ERN amplitude, and diminished response monitoring, indexed by decreased Pe amplitude, reveal emerging cognitive problems in the non-clinical burnout group. Cognitive impairments in burnout subjects relate to both reactive and unconscious (ERN) and proactive and conscious (Pe) aspects of error processing. The results indicate a stronger ‘reactive control mode’ that can deplete resources for proactive control and the ability to actively maintain goals. The analysis refers to error processing and specific task demands, thus should not be extended to cognitive processes in general. The characteristics of ERP patterns in burnout resemble psychophysiological indexes of anxiety (increased ERN) and depressive symptoms (decreased Pe), showing to some extent an overlapping effect of burnout and related symptoms and disorders. The results support the scarce existing data on the psychobiological nature of burnout, while extending and specifying its cognitive characteristics.

## Introduction

Professional burnout is a syndrome that is currently receiving much interest from scientific research and organizational specialists. The concept of burnout is characterized by typical symptoms: psychophysical or emotional exhaustion, depersonalization and diminished professional efficacy (Maslach and Schaufeli, 1993; Maslach et al., 1996, 2001; Maslach and Leiter, 1997, 2004, 2008; Leiter and Maslach, 2004). The processual character of burnout refers to cumulative negative consequences of long-term work-related stress. The core burnout symptoms are exhaustion (associated with a lack of energy, fatigue, and discouragement) and depersonalization/cynicism (associated with withdrawal, lack of motivation and emotional distance with clients, patients or co-workers). These usually lead to a further decrease in personal accomplishment and professional efficacy. However, additional effort and other compensative mechanisms may prevent these consequences (Berggren and Derakshan, 2013; Moser et al., 2013). The sequential process of burnout (e.g., Leiter et al., 2010) implies that the initial state of fatigue and exhaustion may lead to further psychosocial and health consequences.

There are many studies examining the antecedents and prevalence of burnout (for a review see Maslach et al., 2001; Schaufeli et al., 2009), effects on psychosomatic health (Bauer et al., 2006; Melamed et al., 2006; Feuerhahn et al., 2013) and work performance (Beck et al., 2013; Diestel et al., 2013). Despite the fact that burnout has well-documented effects on psychophysical states, a limited number of studies have examined its influence on specific cognitive functions (e.g., van der Linden et al., 2005; Schmidt et al., 2007; Castaneda et al., 2011; Oosterholt et al., 2012, 2014; Deligkaris et al., 2014; Sokka et al., 2014, 2017; Giorgi et al., 2016). Regarding the broader context of the current economic situation (Mucci et al., 2016), the dynamics of workplace changes (Rachiotis et al., 2014; Giorgi et al., 2015), and the prevalence of work-related stress and burnout problems, it is particularly important to study its impact on mental health, well-being and individual functioning.

### Burnout and Cognitive Functions

Based on the assumption that burnout is a stress-related syndrome (Maslach et al., 2001), the research on the consequences of long-term stress on the brain and cognition is particularly interesting (Sapolsky, 1996; Buwalda et al., 2005; Marin et al., 2011). Rönnlund et al. (2013) found that long-term stress is associated with decreased subjective evaluation of memory and cognitive functioning. Nonetheless, there was no significant difference with controls regarding objective tests, such as episodic memory performance, word fluency and block design performance. A systematic review provided by Deligkaris et al. (2014) distinctly showed that burnout is associated with a decline in three main cognitive functions: executive functions, attention and memory (assessed objectively using psychometric tests instead of self-reports). Impaired cognitive functioning is often reported by burnout individuals who complain about attentional and memory problems (van der Linden et al., 2005; Oosterholt et al., 2012; Feuerhahn et al., 2013; Jonsdottir et al., 2013). According to van der Linden et al. (2005), burnout subjects reveal difficulties in voluntary control over attention and the difficulties vary with the severity of burnout symptoms. The latest findings of He et al. (2017) confirmed cognitive impairments in burnout subjects who had lower scores of immediate memory and attention and a lower total score of Repeatable Battery for the Assessment of Neuropsychological Status (RBANS). He et al. (2017) demonstrated that lower cognitive performance in burnout was associated with decreased brain-derived neurotrophic factor (BDNF).

### Error Monitoring

Regarding the core burnout symptoms of emotional exhaustion and depersonalization, cognitive processing in which emotional aspects are involved remains particularly interesting. One well-studied example of this process is error monitoring. Error monitoring is a fundamental component of behavioral regulation that has a significant adaptive function related to signaling and detection of errors that makes it possible to adjust to current and future situations. Compton et al. (2008) have already shown that error monitoring plays a crucial role in an individual’s adaptation, particularly in situations of environmental stress. According to their research, participants who better distinguished between errors and correct responses were less reactive to stressors and exhibited superior emotion regulation in response to stressors in daily life.

Results from electrophysiological studies suggest two main response-locked components of error processing: error-related negativity (ERN) and error positivity (Pe) (Falkenstein et al., 1990, 1991). ERN and Pe reflect neural activity related to action monitoring. Although these two components are observed after errors are committed, they differ in terms of timing and cognitive significance.

The ERN is a negative deflection occurring 0–100 ms after an erroneous response. It is thought to represent a rapid, automatic internal response evaluation mechanism (Unger et al., 2014). Its homolog after correct responses is correct-response negativity (CRN): an event-related potential of similar topography and source of generation, but with much less prominent amplitude. The other event-related potential component associated with the process of response evaluation is Pe: a positive deflection occurring 200–400 ms after an erroneous response and reflecting conscious error recognition (Falkenstein et al., 2000).

The brain structure believed to underlie the process of response evaluation and error detection is the anterior cingulate cortex (ACC), a brain area that is part of the limbic system that has great importance in affect regulation and response selection (Hajcak et al., 2004). The ACC—especially its dorsal part—was found to be the source of the event-related potentials involved in response evaluation (Debener et al., 2005).

The findings of Nieuwenhuis et al. (2001) and Endrass et al. (2007) pointed toward the clarification that ERN and Pe are different processes regarding the level of awareness. While ERN is not modulated by error awareness, Pe is. Pe is described not only as an index of conscious error awareness, but also as motivated attention allocation that enhances behavioral adjustments and may lead to improved performance (Nieuwenhuis et al., 2001; Schroder et al., 2013).

### ERN and Pe in Psychopathology

Studies have suggested that a wide variety of mental or psychological disorders are associated with altered error processing; this is observable in error-related potential (ERP) patterns and is indexed by ERN and Pe components (e.g., Hajcak et al., 2004; Hajcak and Foti, 2008).

#### ERN Component

Patients with obsessive-compulsive disorder, generalized anxiety disorder and major depressive disorder were often characterized by hyperactive early error monitoring, reflected in a larger ERN (for review see Weinberg et al., 2010; Hajcak, 2012; Moran et al., 2017). On the other hand, individuals diagnosed with borderline personality disorder, autism spectrum disorder (for review see Wu et al., 2014) and individuals with substance abuse and impulsive personality characteristics (Hajcak et al., 2004) have decreased ERN amplitude. Moreover, the amplitude of the ERN is known to be influenced by external factors. Examples include: greater amplitude when the task instruction stresses performance accuracy over response speed (Gehring et al., 1993); when there is a prominent discrepancy between a correct and incorrect response (Falkenstein et al., 2000); when the negative affect is induced during the experimental manipulation (Wiswede et al., 2009).

Pfabigan et al. (2013) revealed that a short-lasting subjective state manipulation evoking feelings of helplessness increased ERN amplitude (but did not differentiate the later stages of performance monitoring indicated by Pe amplitude), which proves that such manipulation can modulate some neuronal correlates of action monitoring. This leads to the assumption that context and environmental factors may be considered as independent variables influencing ERP patterns.

In their meta-analysis of the relationship between anxiety and error monitoring, Moser et al. (2013) found a consistent pattern for ERP components for the pathological anxiety indexed by higher ERN amplitude. Anxiety relates to exaggerated error monitoring and is accompanied with typical symptoms including cognitive deficits, impairments in personal functioning, and strategic avoidance behaviors. Regarding the complexity of the studied construct, Moser et al. (2013) indicated two main facets of anxiety: anxious apprehension and anxious arousal. Anxious apprehension is defined by excessive worry and ruminations evoked by indistinct future threats, while anxious arousal is defined by somatic tension and physiological hyperarousal evoked by present and distinct threats. Interestingly, Moser et al. (2013) suggest that increased ERN relates more to anxious apprehension than anxious arousal.

Schroder et al. (2013) point out that depressive symptoms may also be correlated with reduced ERN or show no relation to ERN amplitude. Some data suggests that depression is associated with components that are later than ERN and Pe. Alderman et al. (2016) observed no differences between depressed and control groups in ERN and Pe, but found significant differences in other late ERP components, whose amplitude was higher in depressed groups. Thus, although more evidence supports the increased ERN effect in depression, the outcomes from the aforementioned findings are not conclusive.

#### Pe Component

In a study on anxiety with enhanced concern over mistakes, Tops et al. (2013) observed an increase in Pe amplitude. According to Moser et al. (2013) the relation between the Pe component and anxiety is inconclusive as research variously shows reduced Pe, increased Pe, or revealed no association.

Considering depression and negative affect, the results consistently reveal reduced (e.g., Hajcak et al., 2004; Holmes and Pizzagalli, 2010; Olvet et al., 2010; Schroder et al., 2013) or unaffected (e.g., Alderman et al., 2016) Pe amplitude. Schroder et al. (2013) observed that reduced Pe was related to worse post-error accuracy and stated that reduced Pe in depressed participants may be evidence of worse resource allocation in error trials.

### Error Monitoring in Burnout – ERN and Pe Components

It is already proven that ERP components may be considered as significant markers for many clinical disorders. Burnout, by its correlation with depressive symptoms, negative affect, and anxiety, may also be related to the ERP patterns described above. One important aspect of the aforementioned studies is the clinical character of most studied samples. The question arises if these relationships appear in non-clinical groups. Although Olvet and Hajcak (2008) claim that abnormalities of the ERN are related to relatively stable characteristics, some researchers have already proved that situational changes can modulate ERP patterns. An increase in the ERN amplitude can be observed when the task instruction stresses performance accuracy over response speed (Gehring et al., 1993), or when there is a substantial difference between a correct and incorrect response (Falkenstein et al., 2000). On the contrary, decrease in the ERN amplitude can be observed when presented stimulus is rudimentary (Scheffers and Coles, 2000), due to the fatigue (Scheffers et al., 1999) or in older age (Falkenstein et al., 2001).

Hajcak et al. (2004) suggested that exploration of response monitoring abnormalities in the context of different concepts of psychopathology is an important research area. To the best of these authors’ knowledge, the proposed study is one of the first in which the problem of error monitoring is analyzed in the context of burnout. All the above findings support the idea of electrophysiological indices in burnout groups. The question then arises as to what the specific pattern of ERN and Pe components in burnout groups is. Considering significant correlations between ERP components and clinical disorders, particular attention is paid to the characteristics of the studied group.

### The Severity of Symptoms – Clinical and Non-clinical Burnout

The degree of burnout refers to the range and severity of the symptoms. In the literature, a popular distinction which refers to clinical and non-clinical burnout groups can be found (Oosterholt et al., 2014). The non-clinical burnout group refers to burnout symptoms among employees who can still do their job. The clinical burnout group consists of employees who reveal severe burnout symptoms and who are not able to perform efficiently or are unable to perform their duties at work. The latter group usually requires professional treatment to overcome employees’ problems (Van Dam, 2016). Interestingly, some researchers also indicate the sociocultural context that defines clinical or non-clinical connotations of burnout subgroups. For example, Schaufeli et al. (2009) point out that burnout severity does not only refer to intensity of the consequences of long-term work-related stress, but also to the broader context of the methods of diagnosis and treatment.

Burnout subjects who are drug-free, currently working, and describe themselves as ‘healthy individuals’ without any psychiatric or neurological disorders are assumed in the presented study to refer to a non-clinical group.

Summarizing the above findings, it may be concluded that burnout individuals should reveal impaired response to potentially stressful events. On the basis of existent studies on cognitive impairments, a significant difference between burnout and controls in error processing is expected. Thus, the following main research hypothesis is proposed:

*General hypothesis:* Compared to controls, burnout subjects reveal different ERP patterns in error processing indexed by ERN and Pe amplitudes.

Considering strong burnout correlations with anxiety and depressive symptoms, it can be hypothesized that burnout subjects should reveal increased ERN, as enhanced ERN was observed in most studies in negative affect and depressive or anxiety samples (e.g., Hajcak et al., 2004; Holmes and Pizzagalli, 2010; Moser et al., 2013, respectively).

Regarding the Pe component, it reflects conscious aspects of error monitoring, therefore depletion rather than enhancing would be expected. This would correspond with withdrawal, inhibition, distancing and emotional detachment as symptoms of cynicism and would be in line with some EEG research on depressive symptoms (Hajcak et al., 2004; Holmes and Pizzagalli, 2010; Olvet et al., 2010; Schroder et al., 2013).

Thus, referring to the presented literature review, two specific hypotheses are introduced:

*Hypothesis A*: Burnout subjects reveal enhanced ERN amplitude in error processing.
*Hypothesis B*: Burnout subjects reveal decreased Pe amplitude in error processing.

## Materials and Methods

### Participants

The study was conducted on a group of 80 participants (47 females; mean age = 36.00 years; *SD* = 7.77 years) selected from an initial group of 100 subjects. Participants were excluded on the basis of inconclusive questionnaire results (11 participants), poor EEG data quality (5 participants), and insufficient number of committed errors (4 participants).

This study was carried out in accordance with the recommendations of the APA Ethics Code. All subjects gave written informed consent in accordance with the Declaration of Helsinki. The study protocol was approved by the Bioethics Commission at Jagiellonian University.

Participants were employees with at least 1.5 years of work experience, ranging in age from 25 to 55. They were recruited to the study after completing the Maslach Burnout Inventory – General Survey (MBI-GS; Maslach et al., 1996) and the Areas of Worklife Survey (AWS; Leiter and Maslach, 2004). The presence of burnout symptoms was rechecked with the use of the Link-Burnout Questionnaire (LBQ; Santinello, 2007) at the time of the EEG session.

Subjects were paid for their participation. All of them were reported to be active workers, not to work night shifts, with normal or corrected-to-normal vision. They all described themselves as being right-handed, not suffering from any neurological disorders, not being addicted to psychoactive substances, and not being pregnant.

Based on MBI-GS and AWS results, subjects were divided into two groups: the burnout group and the control group. The burnout group is characterized with high scores on exhaustion (>4) and cynicism (>4) and medium scores on efficacy (<3.5). Additionally, to ensure the work-related context of burnout symptoms, only subjects with low scores in at least three of six AWS scales were selected for the burnout group. Low AWS scores indicate the mismatch between individual and work environment in six work-related areas: workload, control, reward, community, fairness, and values. Furthermore, to control individual characteristics, trait anxiety, neuroticism and depressive symptoms were analyzed on the basis of the State-Trait Anxiety Inventory (STAI; Spielberger, 1989), NEO Five-Factor Inventory (NEO-FFI; Costa and McCrea, 1992), and Beck Depression Inventory (Beck et al., 1988), respectively.

Results of the burnout group (*N* = 40) and demographically matched (including educational level) healthy reference subjects (*N* = 40) were analyzed in the study. The mean age of the burnout group was 37.60; *SD* = 7.27 years (23 females); the mean age of the control group was 34.40; *SD* = 8.02 years (24 females). The descriptive statistics of the burnout and control groups are presented in **Table 1**, including burnout symptoms, work-life areas, anxiety, neuroticism and depressive symptoms and independent *t*-tests with *p*-values.

**Table 1:** The means (M) and standard deviations (SD) for the burnout and control groups on burnout symptoms (exhaustion, cynicism, and efficacy), depressive symptoms, neuroticism, anxiety, work-life areas, and independent-sample *t*-test between burnout and controls.

	BURNOUT (*N* = 40) *M (SD)*	CONTROL (*N* = 40) *M (SD)*	*t*-value (*df* = 78)
**MBI-GS**			
*Exhaustion*	4.32 (0.83)	1.86 (0.70)	–14.34^∗∗∗^
*Cynicism*	4.11 (0.82)	1.44 (0.63)	–16.34^∗∗∗^
*Efficacy*	3.22 (1.11)	4.53 (0.60)	6.58^∗∗∗^
**BDI**			
*Depression*	15.58 (7.18)	4.45 (4.18)	–8.47^∗∗∗^
**NEO**			
*Neuroticism*	27.85 (7.71)	14.98 (6.09)	–8.29^∗∗∗^
**STAI-T**			
*Anxiety*	51.30 (8.19)	39.45 (6.78)	–7.05^∗∗∗^
**AWS**			
*Workload*	2.24 (0.82)	3.23 (0.84)	5.36^∗∗∗^
*Control*	2.42 (0.95)	3.45 (0.67)	5.60^∗∗∗^
*Rewards*	2.36 (0.72)	3.44 (0.65)	6.96^∗∗∗^
*Community*	2.65 (0.99)	3.58 (0.69)	4.89^∗∗∗^
*Fairness*	1.93 (0.63)	3.09 (0.57)	8.66^∗∗∗^
*Values*	2.71 (0.69)	3.57 (0.54)	6.16^∗∗∗^

*^∗∗∗^*p* < 0.001.*

### Task

Participants were presented with an arrow-headed version of the flanker task (Eriksen and Eriksen, 1974). In this paradigm, participants respond to a target presented in the middle and are asked to ignore a simultaneously presented flanker stimuli. The task consists of two stimuli types: congruent, where target and flanker are alike, and incongruent, where target and flankers differ (Van Veen and Carter, 2002). Overall, the Eriksen flanker task is an example of a response-monitoring task and is characterized by good internal consistency and external validity (Olvet and Hajcak, 2008; Foti et al., 2013).

In the current experiment, five horizontally aligned arrowheads were presented in random order on each trial. Incongruent (i.e., < < > < < or > > < > >) and congruent stimuli (i.e., < < < < < or > > > > >) were presented in a 2:1 ratio to enforce the maximal number of committed errors. All stimuli were presented for 200 ms and the submitted response was followed by an ITI that varied from 800 to 1200 ms (mean 1000 ms).

### Experimental Procedure

Participants were seated at a viewing distance of approximately 60 cm and were instructed to press the “1” button with their left index finger if the center arrow was facing to the left and to press the “2” button with right index finger if the center arrow was facing to the right. Responses were submitted using SRbox (Psychological Software Tools Inc.). Moreover, they were told to respond both as quickly and as accurately as possible. Participants performed a practice block containing 20 trials, during which they were presented with external feedback indicating a good, bad or too slow response (the “too slow” screen appeared every time the response was slower than 500 ms, to force participants to respond as quickly as possible during the main experimental block). The actual task consisted of 5 blocks of 60 trials each (300 trials total). Participants were able to control the length of the inter-block interval.

### Psychophysiological Recording, Data Reduction and Analysis

Continuous dense-array EEG data (HydroCel Geodesic Sensor Net, EGI System 300; Electrical Geodesic Inc., Eugene, OR, USA) was collected from a 256 channel EEG at a sampling rate of 250 Hz (band-pass filtered at 0.01–100 Hz with a vertex electrode as a reference) and recorded with NetStation Software (Version 4.5.1, Electrical Geodesic Inc., Eugene, OR, USA). The impedance for all electrodes was kept below 50 kΩ. The offline data analysis was conducted with open source EEGLAB toolbox^1^ (Delorme and Makeig, 2004). Data was digitally filtered to remove frequencies below 0.5 Hz and above 35 Hz. Average reference was recomputed, and bad channels were automatically removed by kurtosis measures with a threshold value of five standard deviations. Next, continuous data was visually inspected in order to manually remove channels or time epochs containing high-amplitude, high-frequency muscle noise, and other irregular artifacts.

Independent component analysis was used to remove artifacts from data. Due to the large number of channels, decomposition of EEG data with the Infomax algorithm was preceded with Principle Component Analysis. Fifty independent components were extracted and visually inspected for each subject. On the basis of the spatiotemporal pattern (Bell and Sejnowski, 1995; Jung et al., 2000), components recognized as blinks, heart rate, saccades, muscle artifacts, or bad channels were removed. Missing channels were interpolated and ICA weights recomputed.

The EEG was segmented for each trial beginning 200 ms before each response onset and continuing for 1000 ms (i.e., for 800 ms following the response), and a 200 ms window from -200 to 0 ms prior to response onset served as the baseline.

The response-related brain activity was measured at the FCz electrode site (average of four fronto-central electrodes), where the error-related activity is maximal. The ERN was evaluated as the mean activity on error trials in the 70–90 ms post-response time-window. The same time-window was applied to epochs containing correct responses to assess the amplitude of CRN. The Pe was defined as the mean activity from 260 to 310 ms following both correct and erroneous responses.

Behavioral measures included both the number of error trials for each subject, as well as accuracy expressed as a percentage of incorrect trials. Moreover, average reaction times (RTs) on error and correct trials were calculated separately. Trials were removed from the analysis if RTs were faster than 200 ms or slower than 1000 ms (1.3% of all trials).

A paired *t*-test was separately performed for the ERN and PE time-windows to assess the significance of the difference between the mean post-response amplitude for error and correct trials.

The post-error mean amplitudes were tested separately for the ERN and the Pe time-window using a *t*-test to assess whether there was a significant difference between the burnout and control group. Similar analyses were performed for mean post-correct amplitudes.

## Results

### Behavioral Results

Accuracy and RT data are presented in **Table 2**. The number of committed errors did not differ between the groups. RTs varied significantly as a function of accuracy [*F*(1,78) = 205.32, *p* < 0.001], with responses significantly faster on error than correct trials. Moreover, there was a significant difference in RT between the burnout and control group [*F*(1,78) = 4.67, *p* < 0.05], with controls responding significantly faster. However, no interaction effect was found. The analysis of post-error and post-correct RT revealed significantly faster responses following errors [*F*(1,78) = 200.93, *p* < 0.001], with no significant difference between the burnout and control group.

**Table 2:** Mean response times and accuracy values for burnout and control group.

	BURNOUT (*N* = 40)	CONTROL (*N* = 40)
**Reaction time (ms)**		
Error trials	396.25 (93.86)	363.43 (63.43)
Correct trials	467.84 (67.08)	435.85 (51.70)
**Post-trial reaction time (ms)**		
Post-error trials	398.84 (92.80)	371.50 (64.90)
Post-correct trials	470.26 (65.80)	438.95 (51.22)
% of errors	4.7%	5.7%

*Standard deviation values are noted in brackets.*

### Psychophysiological Results

**FIGURE 1:**
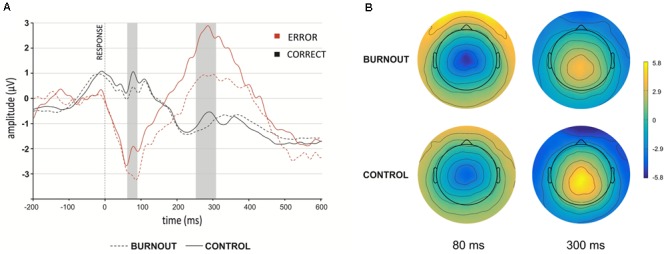
**(A)** Grand-average error-related potential (ERP) waveform after correct (black line) and incorrect (red line) response at the FCz electrode site for burnout and control group **(B)**. Scalp topography of the error-difference wave (error-correct) 80 and 300 ms after the response for burnout and control group. Gray rectangles denote the time-windows selected for the ERP analyses.

**Figure 1A** presents the grand average response-locked ERP waveform at the FCz recording site, comparing correct and error trial waveforms for burnout group and controls. The presented waveforms depict a difference in the post-error activity (red line) for the ERN component (marked by the first gray shadow bar) and the Pe component (marked by the second gray shadow bar) between the burnout (dotted line), and control (solid line) groups. The gray line presents post-correct ERP waveform (dotted line – burnout, solid line – control). **Figure 1B** presents topographic maps for the burnout (top) and control (bottom) groups, depicting voltage differences (in uV) across the scalp for error minus correct responses in the time-window of the ERN (80 ms) and Pe (300 ms). In addition, **Table 3** presents grand-average mean amplitude values of ERN, CRN, and Pe (for both post-error and post-correct trials, respectively) for burnout, control, and total number of participants.

**Table 3:** Grand-average mean amplitude values of error-related potential (ERN), correct-response negativity (CRN), and error positivity (Pe) (for both post-error and post-correct trials, respectively) for both burnout and control groups, and total number of participants.

	Grand-average amplitude in μV (SD)		
	BURNOUT (*N* = 40)	CONTROL (*N* = 40)	TOTAL
***ERN***	-3.12 (2.56)	-2.01 (2.36)	-2.57 (2.51)
***CRN***	0.29 (1.38)	0.87 (1.49)	0.58 (1.46)
***Pe***			
*Post-error trials*	0.89 (2.66)	2.69 (3.72)	1.79 (3.34)
*Post-correct trials*	-1.08 (1.66)	-0.64 (1.72)	-0.86 (1.69)

### The ERN/CRN

**FIGURE 2:**
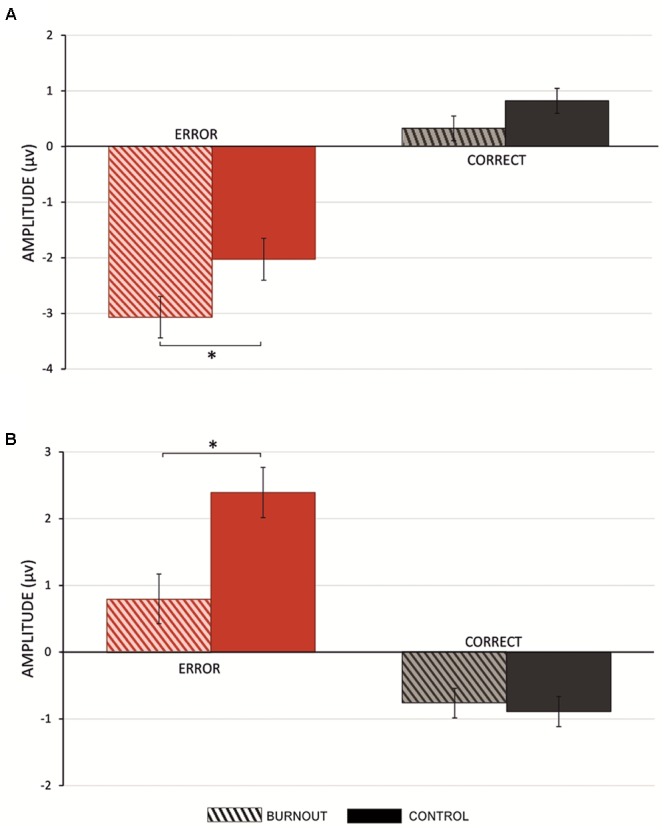
**(A)** Mean amplitudes of post-error (red) and post-correct (gray) response ERP waveforms in a time-window of ERN for burnout and control group. **(B)** Mean amplitudes of post-error (red) and post-correct (gray) response ERP waveforms in a time-window of error positivity (Pe) for burnout and control groups. Vertical bars denote standard errors; asterisks denote statistically significant differences.

**Figure 2A** presents mean amplitude values of the post-error and post-correct response ERP components in the time-window of the ERN/CRN for the burnout and control groups. There was a significant difference between the response-related post-error (*M* = -2.57, *SD* = 2.51) and post-correct (*M* = 0.58, *SD* = 1.46) mean amplitude; *t*(79) = -9.54, *p* < 0.001. Thus, the significantly more negative mean amplitude for the erroneous trials and the mean amplitude for the correct trials account for the ERN and CRN, respectively.

The analysis of a between-groups difference in the mean post-error activity revealed that burned-out participants had a significantly larger ERN, i.e., more negative amplitude (*M* = -3.12, *SD* = 2.56) than the control group (*M* = -2.01, *SD* = 2.36); *t*(78) = 2.02, *p* < 0.05. The amplitude of the CRN did not differentiate the groups [*t*(78) = 1.79, *p* > 0.05] (for reference, see **Figure 2A** and **Table 3**).

### The Pe

**Figure 2B** presents mean amplitude values of the post-error and post-correct ERP components in the time-window of the post-response positivity for the burnout and control groups. In the time-window of post-response positivity, there was a significant difference between the response-related post-error (*M* = 1.79, *SD* = 3.34) and post-correct (*M* = -0.86, *SD* = 1.69) mean amplitude; *t*(79) = 7.57, *p* < 0.001. Thus, the Pe had significantly more positive mean amplitude for erroneous trials than correct ones.

The analysis of a between-groups difference in the mean post-error activity revealed that burned-out participants had a post-error Pe of significantly smaller (i.e., less negative) amplitude (*M* = 0.89, *SD* = 2.66) than the control group (*M* = 2.69, *SD* = 3.72); *t*(78) = 2.02, *p* < 0.05. The amplitude of the post-response activity in the time-window of Pe after correct responses did not differentiate the groups [*t*(78) = 1.1, *p* > 0.1] (for reference, see **Figure 2B** and **Table 3**).

## Discussion

The presented study examined neural mechanisms of error processing among burnout individuals compared to a control group. The results are discussed in the context of behavioral research, ERP studies, as well as theoretical models and conceptions. The findings are also related to important scientific discussion on the overlapping effects of burnout, specific individual traits, and other symptoms and disorders.

Behavioral results revealed a significant difference in RTs, with controls responding significantly faster, but did not show a significant error rate difference between the groups. Therefore, notable behavioral adjustments in the burnout group lead to prolonged RTs on trials subsequent both to correct and erroneous trials, thereby reflecting a more cautious response mode that is consistent with earlier research (Rabbitt, 1966; Ridderinkhof et al., 2004). The effect of post-error slowing is not observed in the presented study. Schroder et al. (2013) concluded that post-error slowing may not be an inherent and adaptive reaction in each error commission situation: specific task context may be an important moderator here. The obtained behavioral results are consistent with many studies on depression (see for review Schroder et al., 2013) that suggest that in relatively simple flanker tasks, post-error performance impairments were not observed.

The presented results indicate that burnout individuals react slower, although no differences were observed on the performance level. The results are consistent with Moser et al.’s (2013) analysis: correlations between ERN and error range were found in only 3 of 37 reported studies; furthermore, in most studies performance level did not differentiate anxious and control groups. Moreover, none of the studies revealed a relationship between anxiety and RT.

The behavioral data is in line with Oosterholt et al.’s (2014) study, which compared three groups: clinical burnout, non-clinical burnout, and healthy control group. Only a mild impairment in cognitive test performance was observed in the clinical burnout group. The impairment was associated with slower RT, but no evidence of impaired cognitive functions was found. Similarly, Diestel et al. (2013) did not observe any differences in performance between exhausted and not exhausted subjects if tasks put low demands on executive control. The significant difference was observable only when a task was associated with high demands on executive control. Diestel et al. (2013) confirmed worse performance among burnout individuals, but only in specific circumstances. Similar tendencies were reported by Österberg et al. (2009). In many cases, only by analyzing particular conditions or certain detailed aspects of individual functioning was is possible to differentiate burnout subjects from controls. Furthermore, as many findings did not reveal a significant performance level difference between burnout/long-term stressed subjects and control groups, the criterion of lower efficacy and impaired performance is not so evident. Therefore, deeper and more detailed analyses could explain the specific problems of cognitive functioning among burnout subjects.

### ERN and Pe Components in Burnout

In terms of ERPs, participants diagnosed with burnout syndrome were characterized by significantly greater ERN amplitude in error trials, suggesting that this stress-related syndrome modulates the first stage of error processing linked with automatic detection of errors (Falkenstein et al., 2000).

The relation between ERN and depression and anxiety is not consistent in existing research; therefore, it is especially desirable to compare results referring to similar tasks. Schroder et al. (2013) compared the results of their study on depressive disorder to other EEG studies. The researchers pointed that their observation of reduced Pe and intact ERN is similar to studies in which a flanker task was used (cf. Olvet et al., 2010). In terms of corresponding task demands, one could conclude that the burnout group can be distinguished from the depressive group on the basis of decreased ERN in error trials.

Regarding the phase of error monitoring, ERN demonstrates the first reflexive reaction, which seems to be exaggerated among burnout subjects. If, as pointed out by Maier et al. (2008), ERN is not linked to error detectability, it may imply that error commission is more significant for burnout subjects and evokes a stronger electrophysiological response.

Gehring et al. (1993) and Olvet and Hajcak (2008) suggested that ERN might relate to the significance of an error as it reflects ongoing evaluation of the response correctness and response conflict. If errors may be considered salient events which evoke emotional and motivational responses, the neural response for such events may be perceived as an index of an individual’s emotional and motivational state. Thus, increased ERN in burnout may be a manifestation of both the significance of committed errors and increased sensitivity to committing errors. It can furthermore be an index of enhanced emotional reaction to erroneous response in burnout subjects.

Increased ERN in burnout may be described in terms of contemporary models of psychopathology (Krueger, 1999) and related to symptoms of internalizing disorder and core personality traits, i.e., neuroticism, negative emotionality, and behavioral inhibition. The dichotomic tendency in ERP changes (Hajcak et al., 2004) refer directly to internalizing and externalizing disorders. Hyperactive error processing indexed by increased ERN may characterize internalizing disorders, while hypoactive error processing indexed by decreased ERN may reflect externalizing disorders.

Significant correlations between burnout and anxiety, depressive symptoms and neuroticism have been revealed in many cited works. Additionally, most of these personality traits correlate with increased ERN (e.g., Amodio et al., 2008). Overall, it may be concluded that both objective and subjective measures support the idea of internalizing the nature of burnout syndrome.

Furthermore, Luu et al. (2000) and Hajcak et al. (2004) argued that increased ERN is not typical of pathological conditions of anxiety or depression; however, it reflects their underlying, core attribute, i.e., negative affect. Taking this perspective, it may be assumed that negative emotionality underlines the problems of impaired error processing in burnout individuals.

Smaller Pe in burnout subjects after erroneous responses implies reduced error awareness and attention allocation. Presumably, it is the basis for maladaptive reactions to failures. As is emphasized by Schroder et al. (2013), the trend of reduced Pe may be linked to diminished ability to adapt after failures, impaired coping strategies and helpless behavior.

If Pe, as much evidence indicates, reflects the conscious awareness of error commission (cf. Nieuwenhuis et al., 2001; Murphy et al., 2012; Schroder et al., 2013), reduced Pe amplitude in burnout groups may be associated with the decreased conscious detection of committed errors and reduced ability to allocate attention in order to minimize the possibility of committing errors in the future.

Pe relates to post-error behavioral adjustment and may be linked to slower or more accurate responses (e.g., Nieuwenhuis et al., 2001; Schroder et al., 2013). The presented findings have revealed no differences in post-error behavioral measures. In this context, lower Pe may be interpreted in relation to diminished awareness of mistakes. The presented findings have not revealed any difference in post-error behavioral measures, so in this context lower Pe may be interpreted in relation to diminished awareness of mistakes. Maintenance of the same level of performance may stem from possible compensatory mechanisms developed by burnout subjects which require greater effort. In more complex and demanding tasks or situations, a decrease in Pe may be reflected by lower efficacy, which is a typical burnout symptom.

### The ‘Hidden Costs’ of Burnout

Some neuroimaging studies (for a review see Berggren and Derakshan, 2013) have revealed that increased neural activity in anxiety was linked to a possible compensatory effort. This helped to explain the maintaining of high performance in a broad range of attentional and memory tests. Moser et al. (2013) suggest that enhanced ERN may be an index of the compensatory effort and greater utilization of processing resources.

All of this leads to the conclusion that not effectiveness but efficiency is essential to the correct understanding of burnout. Although burnout subjects do not differ from control subjects in performance level (especially in non-demanding tasks), the costs invested in action performance may be higher. The ‘effort to effect’ ratio may be inadequate for expectations and needs. The costs of additional effort implemented in task performance may cause a state of imbalance that might subsequently activate defense mechanisms.

### Burnout and Reactive and Proactive Control

The results can also be discussed from the perspective of Braver’s (2012) ‘dual mechanisms of control’ theory. Similar conclusions were drawn by Schroder et al. (2013) in relation to specific Pe patterns in depressed symptoms, and by Moser et al. (2013) regarding specific ERN patterns in anxiety. Braver explains the alternating nature of cognitive control and proposes a dual-mechanism framework. He focuses on the diversity and temporal dynamic of cognitive control processes. The differences between the two aspects of control refer to reactive and proactive control. Reactive control relates to transient, stimulus-driven goal reactivation, while proactive control enables optimal cognitive performance and refers to anticipatory maintenance of goal relevant information (Braver, 2012).

Schroder et al. (2013) speculate that depressive symptoms are associated with problems of the engagement of proactive control. As the presented results in the aspect of Pe amplitude are similar to Schroder et al.’s (2013) findings, it leads to the hypothesis that a similar assumption may arise in the context of burnout. Moreover, regarding hyperactive error processing related to the initial, unconscious stage indexed by increased ERN amplitude, an additional presumption may refer to problems with adequate reactive control in burnout. This is in line with Moser et al. (2013), who emphasize that enhanced ERN is associated with increased transient reactive control and reduced preparatory proactive control. Moser et al. (2013) argue that it may be explained by the compensatory error-monitoring hypothesis. Particularly for anxiety, increased ERN is associated with distracting effects of worry, and a compensatory effort is dedicated to constant reactivation of task goals (enhanced reactive control).

Proactive control is a cognitively demanding process as it relates to active maintenance of goals and rules that facilitate future performance. Reactive control is less effortful: it involves allocating attention to goals and rules as and when required (Braver, 2012). According to Braver, healthy subjects are able to flexibly change between reactive and proactive control modes. Increased ERN in chronically anxious people may indicate a stronger ‘reactive control mode,’ which can deplete resources for proactive control and hence the ability to actively maintain goals.

### Burnout and Individual Traits

Wu et al.’s (2014) work is a rare example in which error processing and ERP components in long-term stress context were studied. Their research is particularly interesting as the stress influence was analyzed in a homogenous group of subjects whose personality trait characteristics did not differentiate the compared groups. Long-term academic stress evoked an increase in Pe amplitude but did not influence ERN amplitude. This might implicate a significant role of trait characteristics as variables determining the ERN component in subjects influenced by long-term environmental stress. Comparing Wu et al.’s (2014) study with presented findings, it may be assumed that enhanced Pe in Wu et al.’s (2014) study reflected higher motivational assessment in the stressed group, while in the presented study enhanced ERN reflects higher emotional response (generally) and decreased Pe (lower motivational assessment), but only in the situation of error commission.

The presented findings may also stem, to some extent, from individual differences in anxiety traits and neuroticism. On the one hand, the differences are well-documented variables that are linked to larger ERN amplitude (Weinberg et al., 2012), while on the other hand they are significant predisposing factors to burnout (Langelaan et al., 2006; Mojsa-Kaja et al., 2015).

Van Dam (2016) point out that burnout overlaps with anxiety and depressive disorders. Bianchi et al. (2015) even concluded that it is questionable if the two distinct entities of burnout and depression should be introduced. Instead, the two main dimensions of burnout—exhaustion and depersonalization—are seen as depressive responses to a negative work-related environment.

The presented findings address the scientific discussion on the differences between burnout and other disorders, such as depression (Brenninkmeyer et al., 2001). On the electrophysiological level, both depressive and burnout individuals differ in the magnitude of ERN amplitude as compared to controls, but only major depressive disorder leads to differences in Pe amplitude (Olvet et al., 2010). Anxiety, another strong burnout correlate, also indicates ERN amplitude differences. Anxious subjects consistently demonstrate increased ERN response throughout the various pieces of research. On the contrary, anxious individuals do not present slower RTss, as was observed in burnout subjects who were significantly slower than controls. Thus, the presented study provides more evidence of the overlapping effect between these disorders, but also introduces important differences: ERP patterns in error processing and behavioral measures (RTs) in burnout resemble neither patterns observed in anxious individuals, nor in depressive ones. In fact, the relationships observed in the burnout group can be described as a combined anxiety-depression pattern.

### Limitations

The limitation of the presented study refers to the causal relationship between burnout and abnormal psychophysiological indices of response monitoring. Hajcak et al. (2004) showed that negative affect was connected with enhanced ERN and reduced Pe, but emphasized that the causal interferences relating negative affect and abnormal ERP patterns required experimental manipulation. Similarly to their assumption, in the presented study it would be possible to state the causal effect if it were possible to observe the influence of change in burnout symptoms (in the sense of level and severity) on psychophysiological responses in the long-term.

Another limitation refers to the difficulty in distinguishing burnout from other closely related characteristics and symptoms such as neuroticism, anxiety or depression. In the presented study, neuroticism, anxiety and depression were highly correlated with burnout; this is consistent with existing data (Langelaan et al., 2006; Mojsa-Kaja et al., 2015; Van Dam, 2016). It is possible to separate subjects with, e.g., comparable levels of depressive symptoms, but this would result in studying non-typical burnout and healthy samples. The distribution of neuroticism, anxiety and depression differs between burnout and healthy samples and much research has proved these interdependencies.

An additional limitation of the presented study is that it relied on self-report measures of burnout and related constructs (such as depression), which have satisfying validity but are not the only means of assessing analyzed stress-related syndrome. Therefore, further research should be enriched by independent diagnosis performed by a clinical psychologist.

When analyzing the individual histories and context determinants of tested burnout subjects, it may be concluded that their state was highly influenced by their work-related environments. Additional measures (AWS, structured interview) were incorporated to find the source of their problems. This made it possible to ensure that work-related stress was at least to some extent a causal factor of deterioration in subjective well-being. Still, there is a question: to what extent? Longitudinal studies could find if individual characteristics and work-related conditions and stressors should be considered in parallel with the well-being of workers (studied with subjective and objective measures). The complexity of possible factors influencing all those relationships, such as subjects’ health, personal history, changing work demands and resources, etc., reveal the difficulties of this study design. Leaving aside individual characteristics, which always play an important role in burnout, methodological issues limit the more precise estimation of the extent of solely work-related stress. Previous research and existing data show that individual characteristics such as neuroticism, anxiety, negative affect and depressive symptoms are associated with burnout, some of them may predispose burnout, and they are close to burnout in some behavioral and neural manifestations.

Thus, it may be concluded that there is no clear distinction between patterns of neural activity underlying burnout syndrome and those associated with, e.g., anxiety or depression. The observed neural activity reflects a subtle cognitive impairment in error processing which is not exclusively related to burnout. Moreover, regarding biographical and contextual analyses it may be assumed that these impairments, at least to some extent, might be a result of work-related long-term stress.

## Conclusion

The obtained results indicate impaired error processing in individuals presenting burnout symptoms; cognitive impairments are indexed by enhanced ERN and decreased Pe amplitude.

This conclusion supports the research hypotheses: compared to controls, burnout subjects reveal different ERP patterns in error processing, indexed by enhanced ERN and decreased Pe amplitudes. The deficits in cognitive control in burnout subjects refer to two phases of error monitoring: error detection (indexed by ERN) and response monitoring (indexed by Pe), which might have a further influence on implementation of adequate behavioral adjustments.

*Error processing among burnout individuals relies more on reactive control, which seems to have a negative influence on proactive control*.

In the context of the presented studies and theories, it may be assumed that two kinds of cognitive impairments are found in burnout groups: inadequate reactive control, which refers to exaggerated reaction to errors, and insufficient proactive control, which is associated with reduced response monitoring and impaired goal-oriented processes. The first impairment is possibly linked to high sensitivity to salient, negative stimulus and anxious apprehension, while the second is associated with diminished cognitive resources to actively plan and monitor further actions.

The conclusions of the presented study relate to error detection and error monitoring and should not be generalized on other aspects of information processing.

The significant difference in the ERN and Pe amplitudes between the burnout and control group were observed only in error trials. The results might suggest that burnout did not influence the phase of conscious recognition and did not modulate the level of action awareness in neutral or positive events (as in the correct trial). Further research could consider other examples of salient events and emotion-related stimuli to verify whether these findings reveal universal cognitive processes of error monitoring in burnout subjects, or are specific task-related correlations.

Changes in ERN characteristics in the burnout group may be analyzed as symptoms or markers of pathological processes with abnormal response monitoring.

In a broad review of functional, neurobiological, and developmental studies, Olvet and Hajcak (2008) concluded that increased error-related brain activity indexed by ERN amplitude is associated with the internalizing dimensions of psychopathology and that neural and information-processing abnormalities may indicate the risk of developing psychopathology. The presented study provides support for the possible underlying mechanisms of the cognitive deficits/impairments in burnout individuals. This may help in understanding worse performance on cognitively demanding tasks that require efficient cognitive processing and extend the knowledge of mechanisms that refer to exhaustion and depersonalization.

When studying ERPs as biomarkers of psychotic disorders, Foti et al. (2013) revealed that task specificity might strongly influence the outcomes and conclusions. This is an important suggestion for future studies: the relations between error monitoring impairments and psychopathological symptoms should be related to specific task demands as important moderators of studied mechanisms.

To sum up, the results of this study hold the promise of identifying ERP as a clinically useful measure for detecting risk of specific cognitive impairments (error monitoring impairments) among burnout individuals and adds to the body of knowledge of cognitive functioning, which might be helpful in providing care for burnout individuals.

## Author Contributions

KG and MG substantial contributions to the conception and design of the work; acquisition, analysis, interpretation of data, drafting the work and revising it critically; final approval of the version to be published; agrees to be accountable for all aspects of the work. JM-K substantial contributions to the conception and design of the work; analysis, interpretation of data, drafting the work and revising it critically; final approval of the version to be published; agrees to be accountable for all aspects of the work. KP: substantial contributions to acquisition, analysis, interpretation of data, drafting the work and revising it critically; final approval of the version to be published; agrees to be accountable for all aspects of the work.

## Conflict of Interest Statement

The authors declare that the research was conducted in the absence of any commercial or financial relationships that could be construed as a potential conflict of interest.

## Footnotes

1 http://sccn.ucsd.edu/eeglab

## References

[B1] Alderman B. L. Olson R. L. Brush C. J. Shors T. J. (2016). MAP training: combining meditation and aerobic exercise reduces depression and rumination while enhancing synchronized brain activity. *Transl. Psychiatry* 6:e726 10.1038/tp.2015.225 PMC4872427 26836414

[B2] Amodio D. M. Master S. L. Yee C. M. Taylor S. E. (2008). Neurocognitive components of the behavioral inhibition and activation systems: implications for theories of self-regulation. *Psychophysiology* 45 11–19. 10.1111/j.1469-8986.2007.00609.x 17910730

[B3] Bauer J. Stamm A. Virnich K. Wissing K. Müller U. Wirsching M. (2006). Correlation between burnout syndrome and psychological and psychosomatic symptoms among teachers. *Int. Arch. Occup. Environ. Health* 79 199–204. 10.1007/s00420-005-0050-y 16258752

[B4] Beck A. T. Steer R. A. Carbin M. G. (1988). Psychometric properties of the beck depression inventory: twenty-five years of evaluation. *Clin. Psychol. Rev.* 8 77–100. 10.1016/j.jpsychires.2013.08.009

[B5] Beck J. Gerber M. Brand S. Pühse U. Holsboer-Trachsler E. (2013). Executive function performance is reduced during occupational burnout but can recover to the level of healthy controls. *J. Psychiatr. Res.* 47 1824–1830. 10.1016/j.jpsychires.2013.08.009 24018104

[B6] Bell A. J. Sejnowski T. J. (1995). An information-maximization approach to blind separation and blind deconvolution. *Neural Comput.* 7 1129–1159. 10.1162/neco.1995.7.6.1129 7584893

[B7] Berggren N. Derakshan N. (2013). Attentional control deficits in trait anxiety: why you see them and why you don’t. *Biol. Psychol.* 92 440–446. 10.1016/0272-7358(88)90050-5 22465045

[B8] Bianchi R. Schonfeld I. S. Laurent E. (2015). Is burnout separable from depression in cluster analysis? A longitudinal study. *Soc. Psychiatry Psychiatr. Epidemiol.* 50 1005–1011. 10.1007/s00127-014-0996-8 25527209

[B9] Braver T. S. (2012). The variable nature of cognitive control: a dual mechanisms framework. *Trends Cogn. Sci.* 16 106–113. 10.1016/j.tics.2011.12.010 22245618 PMC3289517

[B10] Brenninkmeyer V. Van Yperen N. W. Buunk B. P. (2001). Burnout and depression are not identical twins: Is decline of superiority a distinguishing feature? *Pers. Individ. Dif.* 30 873–880. 10.1016/S0191-8869(00)00079-9

[B11] Buwalda B. Kole M. H. Veenema A. H. Huininga M. de Boer S. F. Korte S. M. (2005). Long-term effects of social stress on brain and behavior: a focus on hippocampal functioning. *Neurosci. Biobehav. Rev.* 29 83–97. 10.1016/j.neubiorev.2004.05.005 15652257

[B12] Castaneda A. E. Suvisaari J. Marttunen M. Perälä J. Saarni S. I. Aalto-Setälä T. (2011). Cognitive functioning in relation to burnout symptoms and social and occupational functioning in a population-based sample of young adults. *Nord. J. Psychiatry* 65 32–39. 10.3109/08039488.2010.485328 20500121

[B13] Compton R. J. Robinson M. D. Ode S. Quandt L. C. Fineman S. L. Carp J. (2008). Error-monitoring ability predicts daily stress regulation. *Psychol. Sci.* 19 702–708. 10.1111/j.1467-9280.2008.02145.x 18727786

[B14] Costa P. T. McCrea R. R. (1992). *Revised NEO Personality Inventory (NEO PI-R) and Neo Five-Factor Inventory (NEO-FFI).* Odessa, FL: Psychological Assessment Resources.

[B15] Debener S. Ullsperger M. Siegel M. Fiehler K. von Cramon D. Y. Engel A. K. (2005). Trial-by-trial coupling of concurrent electroencephalogram and functional magnetic resonance imaging identifies the dynamics of performance monitoring. *J. Neurosci.* 25 11730–11737. 10.1523/JNEUROSCI.3286-05.2005 16354931 PMC6726024

[B16] Deligkaris P. Panagopoulou E. Montgomery A. J. Masoura E. (2014). Job burnout and cognitive functioning: a systematic review. *Work Stress* 28 107–123.

[B17] Delorme A. Makeig S. (2004). EEGLAB: an open source toolbox for analysis of single-trial EEG dynamics including independent component analysis. *J. Neurosci. Methods* 134 9–21. 10.1016/j.jneumeth.2003.10.009 15102499

[B18] Diestel S. Cosmar M. Schmidt K. H. (2013). Burnout and impaired cognitive functioning: the role of executive control in the performance of cognitive tasks. *Work Stress* 27 164–180. 10.1080/02678373.2013.790243

[B19] Endrass T. Reuter B. Kathmann N. (2007). ERP correlates of conscious error recognition: aware and unaware errors in an antisaccade task. *Eur. J. Neurosci.* 26 1714–1720. 10.1111/j.1460-9568.2007.05785.x 17880402

[B20] Eriksen B. A. Eriksen C. W. (1974). Effects of noise letters upon the identification of a target letter in a nonsearch task. *Atten. Percept. Psychophys.* 16 143–149. 10.3758/BF03203267

[B21] Falkenstein M. Hohnsbein J. Hoormann J. Blanke L. (1990). “Effects of errors in choice reaction tasks on the ERP under focused and divided attention,” in *Psychophysiological Brain Research* eds Brunia C. H. M. Gaillard A. W. K. Kok A. (Tilburg: Tilburg University Press) 192–195.

[B22] Falkenstein M. Hohnsbein J. Hoormann J. Blanke L. (1991). Effects of crossmodal divided attention on late ERP components. II. Error processing in choice reaction tasks. *Electroencephalogr. Clin. Neurophysiol.* 78 447–455. 10.1016/0013-4694(91)90062-9 1712280

[B23] Falkenstein M. Hoormann J. Christ S. Hohnsbein J. (2000). ERP components on reaction errors and their functional significance: a tutorial. *Biol. Psychol.* 51 87–107. 10.1016/S03010511(99)00031-9 10686361

[B24] Falkenstein M. Hoormann J. Hohnsbein J. (2001). Changes of error-related ERPs with age. *Exp. Brain Res.* 138 258–262. 10.1007/s002210100712 11417467

[B25] Feuerhahn N. Stamov-Roßnagel C. Wolfram M. Bellingrath S. Kudielka B. M. (2013). Emotional exhaustion and cognitive performance in apparently healthy teachers: a longitudinal multi-source study. *Stress Health* 29 297–306. 10.1002/smi.2467 23086898

[B26] Foti D. Kotov R. Hajcak G. (2013). Psychometric considerations in using error-related brain activity as a biomarker in psychotic disorders. *J. Abnorm. Psychol.* 122 520–531. 10.1037/a0032618 23713506

[B27] Gehring W. J. Goss B. Coles M. G. Meyer D. E. Donchin E. (1993). A neural system for error detection and compensation. *Psychol. Sci.* 4 385–390. 10.1111/j.1467-9280.1993.tb00586.x

[B28] Giorgi G. Arcangeli G. Mucci N. Cupelli V. (2015). Economic stress in the workplace: the impact of fear of the crisis on mental health. *Work* 51 135–142. 10.3233/WOR-141844 24594539

[B29] Giorgi G. Mancuso S. Fiz Perez F. Castiello D. Antonio A. Mucci N. (2016). Bullying among nurses and its relationship with burnout and organizational climate. *Int. J. Nurs. Pract.* 22 160–168. 10.1111/ijn.12376 25825025

[B30] Hajcak G. (2012). What we’ve learned from mistakes insights from error-related brain activity. *Curr. Dir. Psychol. Sci.* 21 101–106. 10.1177/0963721412436809

[B31] Hajcak G. Foti D. (2008). Errors are aversive: defensive motivation and the error-related negativity. *Psychol. Sci.* 19 103–108. 10.1111/j.1467-9280.2008.02053.x 18271855

[B32] Hajcak G. McDonald N. Simons R. F. (2004). Error-related psychophysiology and negative affect. *Brain Cogn.* 56 189–197. 10.1016/j.bandc.2003.11.001 15518935

[B33] He S. C. Zhang Y. Y. Zhan J. Y. Wang C. Du X. D. Yin G. Z. (2017). Burnout and cognitive impairment: associated with serum BDNF in a Chinese Han population. *Psychoneuroendocrinology* 77 236–243. 10.1016/j.psyneuen.2017.01.002 28119229

[B34] Holmes A. J. Pizzagalli D. A. (2010). Effects of task-relevant incentives on the electrophysiological correlates of error processing in Major Depressive Disorder. *Cogn. Affect. Behav. Neurosci.* 10 119–128. 10.3758/CABN.10.1.119 20233960 PMC3036946

[B35] Jonsdottir I. H. Nordlund A. Ellbin S. Ljung T. Glise K. Währborg P. (2013). Cognitive impairment in patients with stress-related exhaustion. *Stress* 16 181–190. 10.3109/10253890.2012.708950 22746338

[B36] Jung T. Makeig S. Lee T. Mckeown M. J. Brown G. Bell A. J. (2000). “Independent component analysis of biomedical signals,” in *Proceedings of the 2nd International Workshop on Independent Component Analysis and Blind Signal Seperation* Helsinki 633–644.

[B37] Krueger R. F. (1999). The structure of common mental disorders. *Arch. Gen. Psychiatry* 56 921–926. 10.1001/archpsyc.56.10.921 10530634

[B38] Langelaan S. Bakker A. B. Van Doornen L. J. Schaufeli W. B. (2006). Burnout and work engagement: Do individual differences make a difference? *Pers. Individ. Dif.* 40 521–532. 10.1016/j.paid.2005.07.009

[B39] Leiter M. P. Gascón S. Martínez-Jarreta B. (2010). Making sense of work life: a structural model of burnout. *J. Appl. Soc. Psychol.* 40 57–75. 10.1111/j.1559-1816.2009.00563.x

[B40] Leiter M. P. Maslach C. (2004). “Areas of worklife: a structured approach to organizational predictors of job burnout,” in *Research in Occupational Stress and Well Being: Emotional and Physiological Processes and Positive Intervention Strategies* Vol. 3 eds Perrewé P. Ganster D. C. (Oxford: JAI Press/Elsevier) 91–134. 10.1016/S1479-3555(03)03003-8

[B41] Luu P. Collins P. Tucker D. M. (2000). Mood, personality, and self-monitoring: negative affect and emotionality in relation to frontal lobe mechanisms of error monitoring. *J. Exp. Psychol. Gen.* 129 43 10.1037/0096-3445.129.1.43 10756486

[B42] Maier M. Steinhauser M. Hübner R. (2008). Is the error-related negativity amplitude related to error detectability? Evidence from effects of different error types. *J. Cogn. Neurosci.* 20 2263–2273. 10.1162/jocn.2008.20159 18457501

[B43] Marin M. F. Lord C. Andrews J. Juster R. P. Sindi S. Arsenault-Lapierre G. (2011). Chronic stress, cognitive functioning and mental health. *Neurobiol. Learn. Mem.* 96 583–595. 10.1016/j.nlm.2011.02.016 21376129

[B44] Maslach C. Jackson S. E. Leiter M. P. (1996). *The Maslach Burnout Inventory-General Survey Manual* 3rd Edn. Palo Alto, CA: Consulting Psychologist Press.

[B45] Maslach C. Leiter M. P. (1997). *The Truth About Burnout: How Organizations Cause Personal Stress and What to Do About It.* San Francisco, CA: Jossey-Bass.

[B46] Maslach C. Leiter M. P. (2004). “Stress and burnout: the critical research,” in *Handbook of Stress Medicine and Health* ed. Cooper C. (London: CRC Press) 155–172.

[B47] Maslach C. Leiter M. P. (2008). Early predictors of job burnout and engagement. *J. Appl. Psychol.* 93 498–512. 10.1037/0021-9010.93.3.498 18457483

[B48] Maslach C. Schaufeli W. B. (1993). “Historical and conceptual development of burnout,” in *Professional Burnout: Recent developments in Theory and Research* eds Schaufeli W. B. Maslach C. Marek T. (Washington, DC: Taylor & Francispp) 1–16.

[B49] Maslach C. Schaufeli W. B. Leiter M. P. (2001). Job burnout. *Annu. Rev. Psychol.* 52 397–422. 10.1146/annurev.psych.52.1.397 11148311

[B50] Melamed S. Shirom A. Toker S. Berliner S. Shapira I. (2006). Burnout and risk of cardiovascular disease: evidence, possible causal paths, and promising research directions. *Psychol. Bull.* 132 327–353. 10.1037/0033-2909.132.3.327 16719565

[B51] Mojsa-Kaja J. Golonka K. Marek T. (2015). Job burnout and engagement among teachers – worklife areas and personality traits as predictors of relationships with work. *Int. J. Occup. Med. Environ. Health* 28 102–119. 10.13075/ijomeh.1896.00238 26159952

[B52] Moran T. P. Schroder H. S. Kneip C. Moser J. S. (2017). Meta-analysis and psychophysiology: a tutorial using depression and action-monitoring event-related potentials. *Int. J. Psychophysiol.* 111 17–32. 10.1016/j.ijpsycho.2016.07.001 27378538

[B53] Moser J. S. Moran T. P. Schroder H. S. Donnellan M. B. Yeung N. (2013). On the relationship between anxiety and error monitoring: a meta-analysis and conceptual framework. *Front. Hum. Neurosci.* 7:466 10.3389/fnhum.2013.00466 PMC3744033 23966928

[B54] Mucci N. Giorgi G. Roncaioli M. Perez J. F. Arcangeli G. (2016). The correlation between stress and economic crisis: a systematic review. *Neuropsychiatr. Dis. Treat.* 12 983–993. 10.2147/NDT.S98525 27143898 PMC4844458

[B55] Murphy P. R. Robertson I. H. Allen D. Hester R. O’Connell R. G. (2012). An electrophysiological signal that precisely tracks the emergence of error awareness. *Front. Hum. Neurosci.* 6:65 10.3389/fnhum.2012.00065 PMC3314233 22470332

[B56] Nieuwenhuis S. Ridderinkhof K. R. Blom J. Band G. P. Kok A. (2001). Error-related brain potentials are differentially related to awareness of response errors: evidence from an antisaccade task. *Psychophysiology* 38 752–760. 10.1111/1469-8986.3850752 11577898

[B57] Olvet D. M. Hajcak G. (2008). The error-related negativity (ERN) and psychopathology: toward an endophenotype. *Clin. Psychol. Rev.* 28 1343–1354. 10.1016/j.cpr.2008.07.003 18694617 PMC2615243

[B58] Olvet D. M. Klein D. N. Hajcak G. (2010). Depression symptom severity and error-related brain activity. *Psychiatry Res.* 179 30–37. 10.1016/j.psychres.2010.06.008 20630603

[B59] Oosterholt B. G. Maes J. H. Van der Linden D. Verbraak M. J. Kompier M. A. (2014). Cognitive performance in both clinical and non-clinical burnout. *Stress* 17 400–409. 10.3109/10253890.2014.949668 25089935

[B60] Oosterholt B. G. Van der Linden D. Maes J. H. Verbraak M. J. Kompier M. A. (2012). Burned out cognition—cognitive functioning of burnout patients before and after a period with psychological treatment. *Scand. J. Work Environ. Health* 38 358–369. 10.5271/sjweh.3256 22025205

[B61] Österberg K. Karlson B. Hansen Å. M. (2009). Cognitive performance in patients with burnout, in relation to diurnal salivary cortisol: original research report. *Stress* 12 70–81. 10.1080/10253890802049699 18951245

[B62] Pfabigan D. M. Pintzinger N. M. Siedek D. R. Lamm C. Derntl B. Sailer U. (2013). Feelings of helplessness increase ERN amplitudes in healthy individuals. *Neuropsychologia* 51 613–621. 10.1016/j.neuropsychologia.2012.12.008 23267824 PMC3610020

[B63] Rabbitt P. M. (1966). Errors and error correction in choice-response tasks. *J. Exp. Psychol.* 71 264–272. 10.3758/BF03209754 5948188

[B64] Rachiotis G. Kourousis C. Kamilaraki M. Symvoulakis E. K. Dounias G. Hadjichristodoulou C. (2014). Medical supplies shortages and burnout among greek health care workers during economic crisis: a pilot study. *Int. J. Med. Sci.* 11 442–447. 10.7150/ijms.7933 24688306 PMC3970095

[B65] Ridderinkhof K. R. Ullsperger M. Crone E. A. Nieuwenhuis S. (2004). The role of the medial frontal cortex in cognitive control. *Science* 306 443–447. 10.1126/science.1100301 15486290

[B66] Rönnlund M. Sundström A. Sörman D. E. Nilsson L. G. (2013). Effects of perceived long-term stress on subjective and objective aspects of memory and cognitive functioning in a middle-aged population-based sample. *J. Genet. Psychol.* 174 25–41. 10.1080/00221325.2011.635725 23534095

[B67] Santinello M. (2007). *LBQ Link Burnout Questionnaire.* Florence: Giunti Publishers.

[B68] Sapolsky R. M. (1996). Why stress is bad for your brain. *Science* 273 749–750. 10.1126/science.273.5276.749 8701325

[B69] Schaufeli W. B. Leiter M. P. Maslach C. (2009). Burnout: 35 years of research and practice. *Career Dev. Int.* 14 204–220. 10.1108/13620430910966406

[B70] Scheffers M. K. Coles M. G. H. (2000). Performance monitoring in a confusing world: error-related brain activity, judgments of response accuracy, and types of errors. *J. Exp. Psychol. Hum. Percept. Perform.* 26 141–151. 10.1037//0096-1523.26.1.141 10696610

[B71] Scheffers M. K. Humphrey D. G. Stanny R. R. Kramer A. F. Coles M. G. H. (1999). Error-related processing during a period of extended wakefulness. *Psychophysiology* 36 149–157. 10.1017/S0048577299980307 10194961

[B72] Schmidt K. H. Neubach B. Heuer H. (2007). Self-control demands, cognitive control deficits, and burnout. *Work Stress* 21 142–154. 10.1080/02678370701431680

[B73] Schroder H. S. Moran T. P. Infantolino Z. P. Moser J. S. (2013). The relationship between depressive symptoms and error monitoring during response switching. *Cogn. Affect. Behav. Neurosci.* 13 790–802. 10.3758/s13415-013-0184-4 23797948

[B74] Sokka L. Huotilainen M. Leinikka M. Korpela J. Henelius A. Alain C. (2014). Alterations in attention capture to auditory emotional stimuli in job burnout: an event-related potential study. *Int. J. Psychophysiol.* 94 427–436. 10.1016/j.ijpsycho.2014.11.001 25448269

[B75] Sokka L. Leinikka M. Korpela J. Henelius A. Lukander J. Pakarinen S. (2017). Shifting of attentional set is inadequate in severe burnout: evidence from an event-related potential study. *Int. J. Psychophysiol.* 112 70–79. 10.1016/j.ijpsycho.2016.12.004 27988179

[B76] Spielberger C. D. (1989). *State-Trait Anxiety Inventory: Bibliography* 2nd Edn. Palo Alto, CA: Consulting Psychologists Press.

[B77] Tops M. Koole S. L. Wijers A. A. (2013). The Pe of perfectionism: concern over mistakes predicts the amplitude of a late frontal error positivity. *J. Psychophysiol.* 27 84–94. 10.1027/0269-8803/a000090

[B78] Unger K. Greulich B. Kray J. (2014). “Trick or treat”: the influence of incentives on developmental changes in feedback-based learning. *Front. Psychol.* 5:968 10.3389/fpsyg.2014.00968 PMC4158774 25249989

[B79] Van Dam A. (2016). Subgroup analysis in burnout: relations between fatigue, anxiety, and depression. *Front. Psychol.* 7:90 10.3389/fpsyg.2016.00090 PMC4740380 26869983

[B80] van der Linden D. Keijsers G. P. Eling P. Schaijk R. V. (2005). Work stress and attentional difficulties: an initial study on burnout and cognitive failures. *Work Stress* 19 23–36. 10.1080/02678370500065275

[B81] Van Veen V. Carter C. S. (2002). The timing of action-monitoring processes in the anterior cingulate cortex. *J. Cogn. Neurosci.* 14 593–602. 10.1162/08989290260045837 12126500

[B82] Weinberg A. Olvet D. M. Hajcak G. (2010). Increased error-related brain activity in generalized anxiety disorder. *Biol. Psychol.* 85 472–480. 10.1016/j.biopsycho.2010.09.011 20883743

[B83] Weinberg A. Riesel A. Hajcak G. (2012). Integrating multiple perspectives on error-related brain activity: the ERN as a neural indicator of trait defensive reactivity. *Motiv. Emot.* 36 84–100. 10.1007/s11031-011-9269-y

[B84] Wiswede D. Münte T. F. Goschke T. Rüsseler J. (2009). Modulation of the error-related negativity by induction of short-term negative affect. *Neuropsychologia* 47 83–90. 10.1016/j.neuropsychologia.2008.08.016 18786553

[B85] Wu J. Yuan Y. Duan H. Qin S. Buchanan T. W. Zhang K. (2014). Long-term academic stress increases the late component of error processing: an ERP study. *Biol. Psychol.* 99 77–82. 10.1016/j.biopsycho.2014.03.002 24657630

